# Supplementary material for: Mapping the molecular motions of 5-HT3 serotonin-gated channel by voltage-clamp fluorometry
Source: eLife. 2024 Jun 24;12:RP93174. doi: 10.7554/eLife.93174 (PMC11196107; doi:10.7554/eLife.93174)
Supplement: Supplementary file 1. — Values of τrise obtained from rise time kinetic analyses for current and fluorescence on labeled m5-HT3A mutants. [file elife-93174-supp1.docx]

**Supporting Information**

**Mapping the molecular motions of 5-HT_3_ serotonin-gated channel by Voltage-Clamp Fluorometry**

Laurie Peverini^1,*^, Sophie Shi^1^, Karima Medjebeur^1^, Pierre-Jean Corringer^1,*^.

^1^Institut Pasteur, Université́ Paris Cité, CNRS UMR 3571, Channel-Receptors Unit, Paris, France

*Laurie Peverini ; Pierre-Jean Corringer

**Email :**  laurie_peverini@hotmail.com ; pierre-jean.corringer@pasteur.fr

**This file includes:**

Table S1

**Table S1.** Values of 𝛕_rise_ obtained from rise time kinetic analyses for current and fluorescence on labelled m5-HT_3A_ mutants.

| **Construct** | **Molecule** | **Concentrations**  **(µM)** | 𝛕 **(ms) mean +/-SEM** | **Unpaired t-test (p value)** |
| --- | --- | --- | --- | --- |

| S204C | 5-HT | 10 | I | 1490.106 +/- 226.23 | Ns (0.0801) |
| --- | --- | --- | --- | --- | --- |
|  |  |  | F | 2720.210 +/- 602.472 |  |
|  |  | 50 | I | 860.925 +/- 115.317 | Ns (0.0556) |
|  |  |  | F | 1607.027 +/- 332.607 |  |

| S204C | mCPBG | 10 | I | 780.395 +/- 68.012 | * (0.0331) |
| --- | --- | --- | --- | --- | --- |
|  |  |  | F | 1014.284 +/- 50.880 |  |
|  |  | 50 | I | 603.974 +/- 116.549 | Ns (0.4805) |
|  |  |  | F | 751.475 +/- 157.765 |  |

| S204C + N101K | 5-HT | 50 | I | 13744.483 +/- 1207.601 | **** (<0.0001) |
| --- | --- | --- | --- | --- | --- |
|  |  |  | F | 2150.838 +/- 260.847 |  |
|  |  | 100 | I | 9972.073 +/- 1247.551 | **** (<0.0001) |
|  |  |  | F | 2040.467 +/- 177.917 |  |

| S204C + N101K | mCPBG | 50 | I | 1778.377 +/- 148.304 | ** (0.0036) |
| --- | --- | --- | --- | --- | --- |
|  |  |  | F | 1151.664 +/- 74.421 |  |
|  |  | 100 | I | 1387.618 +/- 157.334 | * (0.0206) |
|  |  |  | F | 919.538 +/- 65.337 |  |

| I160C/Y207W | 5-HT | 50 | I | 5754.586 +/-  1010.8129 | **(0.0061) |
| --- | --- | --- | --- | --- | --- |
|  |  |  | F | 2181.044 +/-  371.452 |  |
|  |  | 100 | I | 5989.563 +/-  816.874 | *** (0.0008) |
|  |  |  | F | 1828.336 +/-  202.146 |  |

| I160C/Y207W | mCPBG | 10 | I | 2285.706 +/- 393.976 | Ns (0.8483) |
| --- | --- | --- | --- | --- | --- |
|  |  |  | F | 2173.099 +/- 420.552 |  |
|  |  | 100 | I | 921.791 +/-0216.664 | Ns (0.0508) |
|  |  |  | F | 2242.328 +/- 568.780 |  |

| V106C/L131W | 5-HT | 1 | I | 7868.196 +/- 1860.805 | Ns (0.2294) |
| --- | --- | --- | --- | --- | --- |
|  |  |  | F | 4928.317 +/- 1388.095 |  |
|  |  | 10 | I | 1669.049 +/- 212.492 | Ns (0.1258) |
|  |  |  | F | 1209.388 +/- 174.794 |  |

| V106C/L131W | varenicline | 1 | I | 8099.307 +/- 2081.888 | Ns (0.0755) |
| --- | --- | --- | --- | --- | --- |
|  |  |  | F | 2669.500 +/- 616.985 |  |
|  |  | 100 | I | 1320.949 +/- 215.924 | Ns (0.5363) |
|  |  |  | F | 1120.105 +/- 227.454 |  |

| R219C/Y140W | 5-HT | 1 | I | 1884.998 +/- 323.988 | * (0.0342) |
| --- | --- | --- | --- | --- | --- |
|  |  |  | F | 2936.035 +/- 283.908 |  |
|  |  | 10 | I | 619 +/- 61.883 | ** (0.0080) |
|  |  |  | F | 1490 +/- 267.086 |  |
|  |  | 50 | I | 778.830 +/-156.135 | ns (0.0764) |
|  |  |  | F | 1708 +/- 453.706 |  |

| R219C/Y140W | varenicline | 1 | I | 2417 +/- 272.127 | Ns (0.0575) |
| --- | --- | --- | --- | --- | --- |
|  |  |  | F | 4623.726 +/- 957.392 |  |
|  |  | 10 | I | 544.917 -+/ 32.752 | ** (0.0025) |
|  |  |  | F | 2502.016 +/- 451.889 |  |
|  |  | 100 | I | 397.615 +/- 59.082 | * (0.0336) |
|  |  |  | F | 1825.950 +/- 554.506 |  |

| R219C/Y140W  + N101K | 5-HT | 1 | I | 3467.478 +/- 764.973 | Ns (0.1853) |
| --- | --- | --- | --- | --- | --- |
|  |  |  | F | 5909.440 +/- 1536.925 |  |
|  |  | 10 | I | 1721.749 +/- 274.859 | * (0.0111) |
|  |  |  | F | 3199.143 +/- 433.522 |  |
|  |  | 100 | I | 1135.772 +/- 186.094 | Ns (0.3671) |
|  |  |  | F | 1386.235 +/- 193.816 |  |

| R219C/Y140W  + N101K | varenicline | 10 | I | 2914.184 +/- 192.065 | Ns (0.0851) |
| --- | --- | --- | --- | --- | --- |
|  |  |  | F | 6042.400 +/- 1580.925 |  |
|  |  | 100 | I | 2707.839 +/- 755.202 | Ns (0.5815) |
|  |  |  | F | 3448.594 +/- 1045.416 |  |
